# Circadian reinforcement therapy in combination with electronic self-monitoring to facilitate a safe post-discharge period of patients with depression by stabilizing sleep: protocol of a randomized controlled trial

**DOI:** 10.1186/s12888-019-2101-z

**Published:** 2019-04-25

**Authors:** Signe Dunker Svendsen, Anne Sofie Aggestrup, Lasse Benn Nørregaard, Philip Løventoft, Anne Præstegaard, Konstantin V. Danilenko, Mads Frost, Ulla Knorr, Ida Hageman, Lars Vedel Kessing, Klaus Martiny

**Affiliations:** 1 Copenhagen Affective Disorder research Center (CADIC), New Interventions in Depression (NID) group, Psychiatric Center Copenhagen, University of Copenhagen, Rigshospitalet, Copenhagen, Denmark; 2 Institute of Physiology and Basic Medicine, Novosibirsk, Russia; 3 Monsenso Aps, Rued Langgaardsvej 7, 2300 Copenhagen S, Denmark; 4 Mental Health Services, Capital Region of Denmark, Copenhagen, Denmark

**Keywords:** Major depressive disorder, Sleep, Light, Electronic self-monitoring, Clinician feedback loop, Psychoeducation, Chronotherapeutics, Outpatient treatment

## Abstract

**Background:**

The transition phase from inpatient to outpatient care for patients suffering from Major Depressive Disorder represents a vulnerable period associated with a risk of depression worsening and suicide. Our group has recently found that the sleep-wake cycle in discharged depressive patients became irregular and exhibited a drift towards later hours, associated with worsening of depression. In contrast, an advancement of sleep phase has earlier been shown to have an antidepressant effect. Thus, methods to prevent drift of the sleep-wake cycle may be promising interventions to prevent or reduce worsening of depression after discharge.

**Methods:**

In this trial, we apply a new treatment intervention, named Circadian Reinforcement Therapy (CRT), to patients discharged from inpatient psychiatric wards. CRT consists of a specialized psychoeducation on the use of regular time signals (zeitgebers): daylight exposure, exercise, meals, and social contact. The aim is to supply stronger and correctly timed zeitgebers to the circadian system to prevent sleep drift and worsening of depression. The CRT is used in combination with an electronic self-monitoring system, the Monsenso Daybuilder System (MDB). By use of the MDB system, all patients self-monitor their sleep, depression level, and activity (from a Fitbit bracelet) daily. Participants can inspect all their data graphically on the MDB interface and will have clinician contact. The aim is to motivate patients to keep a stable sleep-wake cycle. In all, 130 patients referred to an outpatient service will be included. Depression rating is blinded. Patients will be randomized 1:1 to a *Standard group* or a *CRT group*. The intervention period is 4 weeks covering the transition phase from inpatient to outpatient care. The primary outcome is score change in interviewer rated levels of depression on the Hamilton Depression Rating Scale. A subset of patients will be assessed with salivary Dim Light Melatonin Onset (DLMO) as a validator of circadian timing. The trial was initiated in 2016 and will end in 2020.

**Discussion:**

If the described intervention is beneficial it could be incorporated into usual care algorithms for depressed patients to facilitate a better and safer transition to outpatient treatment.

**Trial registration:**

Posted prospectively at ClinicalTrials.gov at February 10, 2016 with identifier NCT02679768.

## Background

Major Depressive Disorder (MDD) constitutes a considerable burden for the individual patient and for society, ranking highest on the WHO list of diseases that estimate Disability-Adjusted Life Years (DALY) [1, 2]. MDD is common with a global prevalence of approx. 3–6% [3, 4]. The most severely depressed patients are admitted to psychiatric inpatient wards. Even though patients improve substantially during their stay they are usually still not fully recovered when discharged [5, 6], leaving them at risk of depression worsening, and consequent readmission [7–9]. The suicidal risk in recently discharged patients with MDD has also been found to be markedly increased [10]. Thus, newly discharged patients constitute a very vulnerable group.

The presence of substantial depressive symptoms at discharge necessitates immediate outpatient follow-up treatment. Danish guidelines recommend that the transition period between inpatient and outpatient treatment should be as short as possible. It is also mandatory, according to Danish National quality standards, for all patients to have an appointment with a relevant outpatient health service before discharge [11]. Patients on inpatient psychiatric wards join a predictable and structured milieu with regular meals, physical activities, sleep schedules, medication administration, and round-the-clock care. The transition from inpatient to outpatient status is reported by many patients to be difficult due to the shift from a protective and structured supportive hospitalization environment to being left more to themselves [12].

To this end, there is an urgent need for new tools and treatment methods to be implemented to support patients in this transition.

### Electronic self-monitoring

In recent years, an increasing number of studies have been performed using electronic self-help interventions for depression and anxiety disorders. In general, research points to electronic self-help interventions as useful, and in some cases with efficacy equal to face-to-face therapy [13]. However, for *clinical* depression, therapist-assisted treatment has been shown to be superior to predominantly self-help computer-based cognitive and behavioral interventions [14]. In depression, low education level has been found to increase the risk of symptom deterioration when using these interventions [15]. Adherence seems to be quite high, but dependent on disease severity, treatment length and chronicity [16].

In our previous feasibility study, we developed an electronic monitoring system Daybuilder (now Monsenso Daybuilder = MDB) for use with patients suffering from MDD [6]. The system was found to be reliable and easy to use, even for patients recently treated with Electroconvulsive Treatment. Patients had a high adherence rate and were satisfied with the usability of the program with a System Usability Score of 86.2 (SD = 9.7) (100 as maximum). This system is now used in the present study with some minor alterations: a possibility of entering daily registration of activity (from a Fitbit bracelet), graphical display of sleep instability, text fields to enter a qualitative description of sleep quality, and text fields to enter any used measures to control the sleep-wake cycle. Furthermore, the graphical interface has been improved for better clarity of sleep and mood patterns.

### Sleep, circadian rhythms, and zeitgebers

The human circadian clock, residing in the suprachiasmatic nuclei (SCN) of the hypothalamus, has an intrinsic period averaging 24.2 h [17]. To synchronize with the astronomical 24-h day the period is continually adjusted through external zeitgebers stimuli (external synchronization). Zeitgebers are stimuli capable of synchronizing circadian rhythms such as sleep, melatonin, temperature, heart rate, and blood pressure to provide a stable 24-h rhythm (entrainment). The strongest zeitgeber is light [18], while food [19], exercise [20], and social contact [21], although considered as weaker zeitgebers, can probably also modulate the circadian phase adjustment in humans. Social zeitgebers (e.g. work schedules) act directly or indirectly as they influence the timing of meals, sleep, exercise, and outdoor light exposure. The effect of zeitgeber stimuli is dependent on their intensity and temporal distribution during the 24-h sleep-wake cycle. This has been most extensively described for light where a Phase Response Curve (PRC) [22] shows the advancing or delaying effect of light on the circadian clock dependent on the time of day. Entrainment by light is mediated by the retina, mainly through the intrinsic photoreceptive Retinal Ganglion Cells (ipRGCs) to the SCN and to other brain regions [23, 24].

The setting of peripheral clocks, for example in liver and muscle cells [25], are orchestrated from the SCN in a hierarchical way and influenced by sleep quality, probably mediated through light exposure at night [26]. The exact biological mechanism underlying the synchronization between the SCN and peripheral clocks is not fully understood but is supposed to work through the sympathetic nervous system and through humoral signals such as hormones (glucocorticoids) and cytokines [27]. Recently, we have become aware that peripheral rhythms can be out of synchrony with each other, and thus also out of sync with the orchestrating SCN signals (internal desynchronization) [28]. In depression, there is clear evidence of circadian and seasonal rhythms dysfunction [29], clinically seen as early morning awakening and associated diurnal variation in mood [30], as an advancement of REM sleep within the sleep period [31], as a seasonal recurrence of depression, and as circadian and seasonal variation in neurotransmitters [32, 33]. Desynchronization between the rhythm of the peripheral body clocks, for instance in muscle and liver cells, can be brought about by changes in the timing of exercise [34], meals [35], social activity [36], and maybe sleep [37].

External and internal desynchronization can thus be caused by inappropriate strength and timing of zeitgeber signals due to depression-related alterations in circadian behavior. This includes eating at night, avoiding daylight, light at night, or social isolation with alterations in sleep schedule [38].

Sleep disturbances are present in 50–90% of patients with depression [39]. The biological basis for sleep disturbances in depressed patients are not fully elucidated, but there is evidence for a circadian component such as misaligned morning cortisol increase [40], diurnal variation in mood with evening improvement, staying up late [41], and even inappropriate light habits [42]. Forced changes in the timing of the sleep-wake cycle are often linked to corresponding changes in depression severity. Thus, a sleep-phase delay has been shown to worsen depression [43], and sleep phase advance and sleep deprivation have a well-established antidepressant effect [44, 45]. Furthermore, daytime naps induce hour-long mood drops in a large proportion of depressed patients [46]. Our usability study, using electronic monitoring that documented sleep patterns over a four-week period in patients recently discharged from a mental health hospital, found a day-to-day highly variable sleep-wake cycle with gradually delayed sleep and unstable mood [6].

Therefore, enforcement and timing of zeitgebers with a focus on the regulation of the sleep-wake cycle present itself as a possible new treatment method. In this study we will help patients to attain a time-structured environment with regularly timed zeitgeber stimuli: daylight exposure, exercise, meals, and social contact, to sustain a stable sleep cycle. We have coined this newly proposed intervention: Circadian Reinforcement Therapy (CRT).

## Objectives

The aim of this study is to prevent worsening of depression in patients with MDD after discharge from inpatients psychiatric wards. We hypothesize that the CRT intervention will help patients to recover faster and reduce the likelihood of relapse in the period after discharge.

## Design

The trial protocol is reported according to the SPIRIT statement of randomized trials of nonpharmacologic treatment [47]. The trial is a four-week, randomized, controlled, single-blind, parallel-group trial with a balanced allocation ratio (1:1) of adult patients diagnosed with MDD. Patients will be randomized to either a) a *Standard group* consisting of electronic self-monitoring and usual care at an intensive outpatient service (IOS), or to b) a *CRT group* using the CRT method with electronic monitoring and usual care at the IOS. Patients are psychometrically assessed at baseline and after 4 weeks. The trial investigates the possible effect of CRT on depression. All patients will self-monitor with the MDB system and receive usual care at the IOS. The design of the control group (*Standard group*) was chosen based on the necessity for the intended sample at IOS to have access to qualified and adequate treatment. Thus, placebo treatment was not an option. As we were interested in the isolated effect of the CRT intervention, we opted that both study groups should use the MDB system and receive treatment at the IOS.

## Methods (participants, interventions, and outcomes)

### Study setting

The first patient was enrolled in the study in September 2016 and currently (April 2019), a total of 64 patients have been included. With an expected inclusion rate of 4 patients per month, last patient last visit will be reached July 2020.

The study is conducted at the IOS, a service receiving depressed patients newly discharged from inpatients wards. Treatment is performed by a multidisciplinary team of psychiatrists, nurses, a psychologist, a physiotherapist, and a social worker and includes consultation, medication, psychoeducation, cognitive behavioral therapy, body awareness therapy, and physical exercise.

### Eligibility criteria

All patients referred to the IOS will be screened for eligibility and invited to take part in the study. The eligibility criteria were chosen to represent a broad sample of the patient group to maximize the generalizability of the study results. Eligibility criteria are: participants must be 18 years or older, must have a DSM-IV diagnosis of MDD, and must have given informed consent. Exclusion criteria are patients at risk of suicidal behavior (corresponding to a score of 2 or above on the Hamilton depression rating scale item 3), abuse of alcohol or illegal substances or comorbid dementia or other brain disorders that might hamper the use of the MDB system, bipolar disorder, and psychotic depression. The eligibility will be evaluated through case files and from interviews with the patients.

### Interventions

#### Monsenso Daybuilder and Fitbit description

**Fig. 1:**
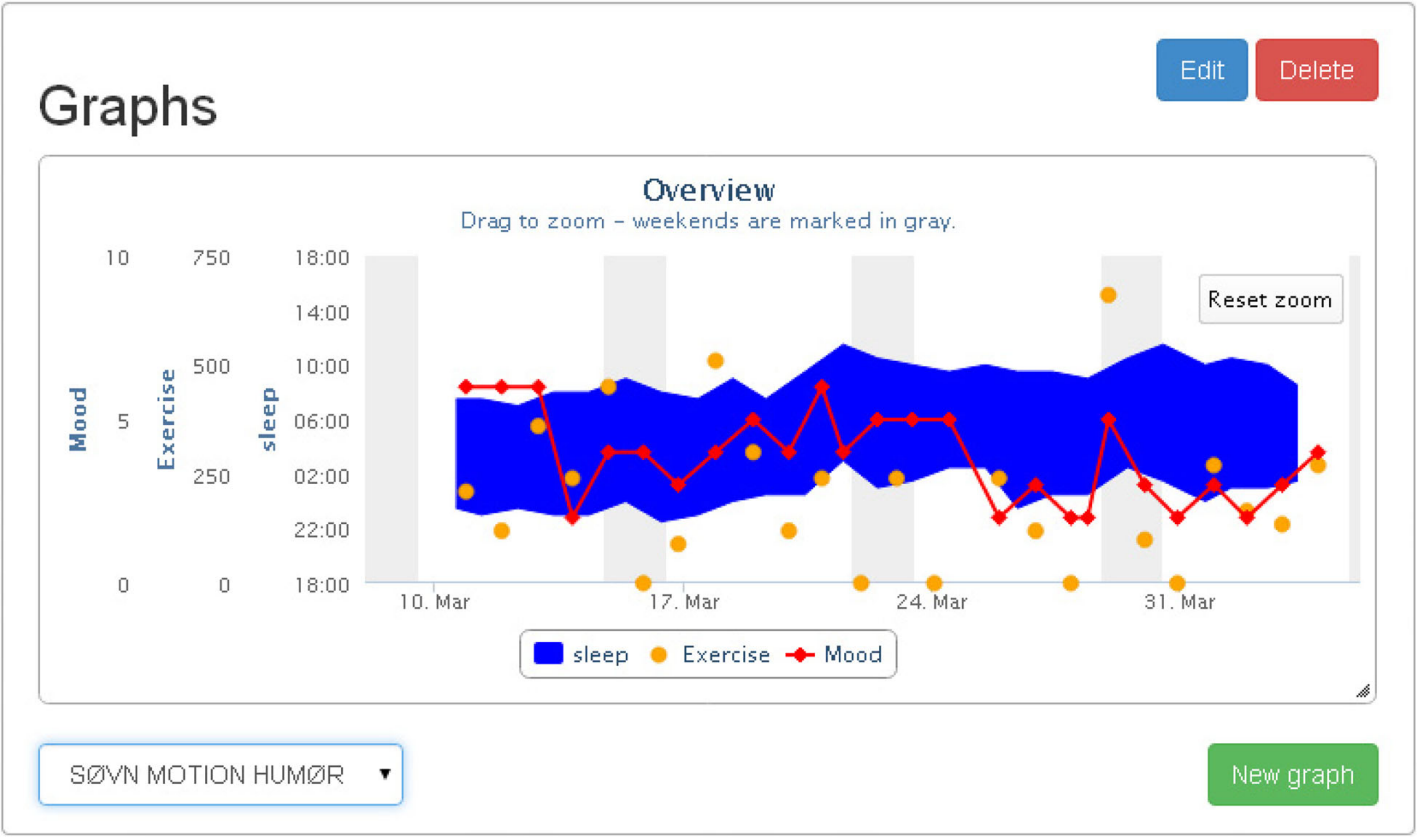
An example of a graphic presentation in the Monsenso Daybuilder System, that can be viewed simultaneously by the patient and the clinician. In this example only mood, sleep and activity are shown. Sleep is visualized as a blue ribbon with the lower border representing sleep-onset and the upper border wake-up time. Mood is shown as a red line with diamond symbols with scores from zero to 10 (10 = best). Activity (=Exercise) is shown as yellow dot symbols representing accumulated 24-h counts measured by a Fitbit charge HR bracelet

The MDB system has been specifically developed for patients suffering from depression in a joint collaboration between software engineers, psychiatrists, and patients [48]. The system can be accessed from all devices using an internet browser. Participants are provided with an iPad and a Fitbit bracelet (version Charge HR). Participants will enter the Fitbit count into the MDB system where it will be displayed along with the other registrations. Each participant is allocated a unique username and password. Participants get access to registrations through a patient interface and investigators through a separate clinician interface. Both interfaces display a similar graphical representation of entered data (see Fig. 1). Patients will be asked to self-assess their sleep and mood twice daily: 0–30 min after waking up, and 0–30 min before going to sleep – and enter it into the MDS.

Morning self-assessments: sleep onset, wake-up time, sleep quality score, number of nighttime awakenings, unlimited qualitative description of their sleep episode, and morning depression severity.

Evening self-assessments: daytime naps with timing and duration of each nap, activity from the Fitbit (the Fitbit resets the activity count at midnight), medication compliance (yes or no), evening depression severity, depression severity as an average of the last 24 h, and unlimited qualitative description of zeitgeber strategies that they have been using for the last 24 h in order to keep a stable sleep-wake cycle (*CRT* group only). Regarding depression severity, patients are instructed to evaluate their depression based on the DSM-IV diagnostic items for MDD. These item descriptions are written on the interface next to the data entry tab: “depressed mood, diminished interest, significant weight or appetite changes, insomnia or hypersomnia, agitation or retardation, loss of energy or tiredness, feelings of worthlessness or guilt, difficulties in concentrate, and suicidal ideations”. All quantitative parameters are scored from zero to 10 (10 is best).

The Fitbit bracelet enables the collection of activity and heart rate. These will be investigated as a predictor for antidepressant response and as markers of the circadian rhythm.

#### Procedures for the Standard group

After an introduction to the MDB system and the Fitbit bracelet, participants will enter data into the system daily in a four-week period. Participants will receive a weekly telephone call from the investigator evaluating (a) experience with the MDB system, (b) suicidal ideations, (c) side effects of medications, (d) worsening of depression, and (e) compliance with data entry. Patients will be reminded to contact either the IOS or the psychiatric emergency unit if they experience aggravation in depressive symptoms.

#### Procedures for the CRT group

Participants in the *CRT group* will enter data into the MDB system, as in the standard group, but an extra module is added to the system, using a graphical depiction of a manometer measuring day-to-day variation in sleep onset, and another manometer measuring changes in sleep midpoint. The manometers serve as a psychoeducational tool for the patients to spot and adjust changes in their sleep timing.

**Fig. 2:**
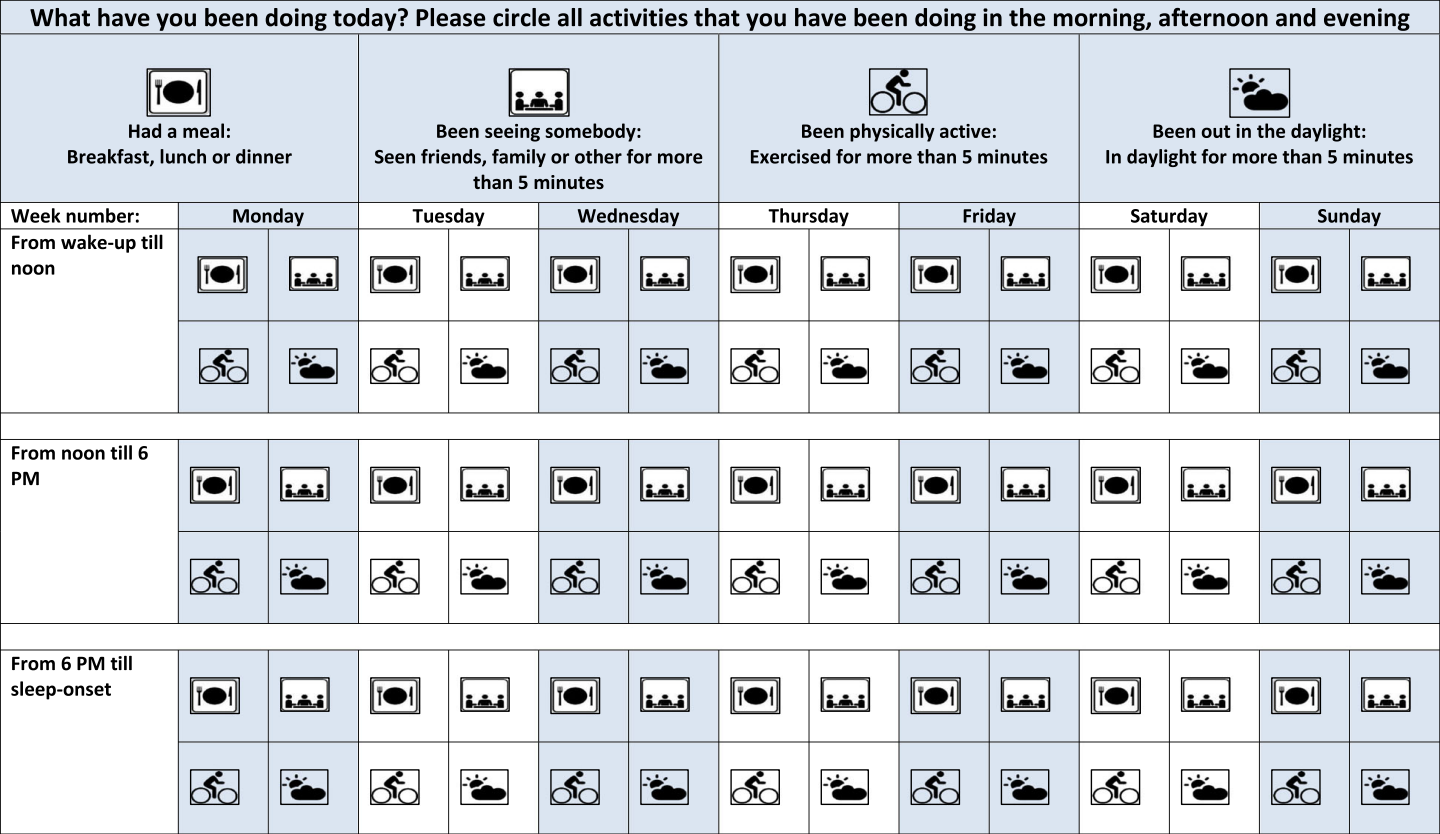
The Chrono schedule used to implement Circadian Reinforcement Therapy (CRT). Patients register daily timing of meals, social interaction, exercise and daylight exposure

The CRT intervention also includes an individual CRT psychoeducational session at baseline and in one of the next two subsequent days, and with booster sessions at the weekly telephone calls. The initial two CRT sessions are provided by the investigator as an individual 1-h session including an introduction to the CRT principles using a full written CRT guide, and a leaflet version, focusing on how participants can incorporate adequate and timed zeitgeber stimuli into their daytime activities regarding daylight exposure, exercise, meals, and social contact. The investigator then helps the participant to fill in a schedule called “basic strategy for daily structure” and provides the participant with a paper and pen diary “The Chrono schedule” where the daily timing of daylight exposure, exercise, meals, and social contact must be noted for the morning, afternoon and evening time-period (see Fig. 2). Finally, the investigator helps the participant to formulate and write down three problems, within the CRT area, they want to work with, in the following 4 weeks, for example: how to achieve an earlier bedtime, how to achieve more daylight, and how to achieve more regular meals.

The Fitbit will be set to a goal of 4000 steps, alerting with a vibration when this is reached, to nudge participants to be more active.

The weekly telephone calls will cover the same elements as in the *Standard group*, supplemented with a discussion of management of the three identified problems, patterns in the Chrono schedule, and in the graphical representation of self-monitored data. This will enable a discussion on how to strengthen zeitgeber stimuli and advance the sleep period and point out any patterns in the graphical display between for example delayed sleep and mood worsening.

Participants’ data registrations, in the MDB system, will be evaluated daily (Monday to Friday) by the investigator. Patients will be contacted if data is missing or if the registrations show predefined trigger signs of aggravation of depression or unstable sleep patterns (described in Table 1).

**Table 1:** Triggers for clinicians telephone call based on inspecting of daily self-assessments in the Monsenso Daybuilder system

Parameter	Evaluation criteria for feedback
Sleep (one of these)	✓More than 10 or less than 4 h sleep a night
	✓A drop-in sleep quality of 4 or above (from 8 to 4 e.g.)
	✓Variation of 3 h or more between two adjoining days in wake-up time or sleep onset
	✓More than 3 awakenings a night for two nights
	✓Late sleep for 3 days – with sleep onset after midnight and wake up time after 9 am
Depression severity (on of these)	✓Drop in daily depression score of 3 or more (e.g. from 8 to 5)
	✓A daily depression score of 2 or less
	✓Morning depression score of 2 or less
Medication	✓More than 1 day without medication registration
Activity	✓More than 1 day without activity registration

#### Concomitant care

All participants will be following the standard IOS treatment program as described above.

### Outcomes

#### Diagnostic measures

The Mini International Neuropsychiatric Interview (M.I.N.I.) 4.0 (Danish version) is used for diagnosis. This is a short, structured diagnostic interview based on the DSM-IV diagnostic system, and will be conducted to confirm depression diagnosis, assess comorbidity, and any contraindications for participation in the trial [49]. Investigators are certified in the use of the M.I.N.I. instrument.

#### Psychometrics and sociodemographics

Sociodemographic data are collected at baseline together with a treatment outcome expectancy rating. Psychometric assessments are performed at baseline and at the endpoint visit. Rating window is set as last 3 days for all instruments except for the Pittsburgh Sleep Quality Index (PSQI) that covers the last month. The severity of depression is assessed by the Hamilton Depression Rating scale, 17 item version, which includes the core symptoms of depression (HAM-D_6_), and the Bech-Rafaelsen Melancholia Rating Scale (MES) focusing more on the cognitive symptoms of depression [50, 51]. The Hamilton rater is blinded to treatment allocation and is trained in the use of the HAM-D_17_ by repeated groups ratings with co-researchers. Self-assessed depression severity is measured by the Major Depression Inventory (MDI), a 10-item questionnaire covering both the ICD-10 and the DSM-IV diagnostic criteria for depression [52]. The response categories of each item are from “at no time” (0) to “all the time” [5], with a score range from 0 (zero) to 50 and a cut-off score of 21 for mild depression, 26 for moderate depression and 31 for severe depression. The WHO-5 Wellbeing index is used as a measure of Quality of life [53]. The WHO-5 consist of 5 items, each rated from “all of the time” (5) to “at no time” (0). The raw score is calculated by summing items multiplicated by four, giving a score range from 0 to 100, where 0 is worst possible and 100 is the best possible quality of life. The Morningness-Eveningness Questionnaire (MEQ) is a 19 items self-assessed questionnaire constructed to assess chronotype. A score below 42 indicates “evening type” and a score above 58 indicates “morning type”; and a score between 42 and 58 indicates “intermediate type” [54]. Sleep quality is assessed by the PSQI containing 11 self-reported items. These items are transformed into 7 “component scores” , and a “global score”. The components scores are “Subjective sleep quality (1)”, “Sleep latency (2)”, “Sleep duration (3)”, “Sleep efficiency (4)”, “Sleep disturbances (5)”, “Sleep medication (6)” and “Daytime dysfunction (7)”. A “Global Score” of 5 or above indicates poor sleep quality [55]. The System Usability Score (SUS) is used to evaluate the usability of the MDB system [56].

#### Biochemical assessments

The Dim Light Melatonin Onset (DLMO) will be determined by saliva sampling every 45 min in the evening from 5 h before expected bedtime until 1 h after expected bedtime at baseline and endpoint (6 samples at each assessment day). The patient will collect samples at home according to a delivered 1-page guideline. The important rule from this guideline is to wear orange goggles (provided by investigators) for the entire 6-h episode of saliva sampling (to prevent suppression of melatonin secretion by blue light). The samples will be refrigerated and kept frozen until the melatonin assay. All samples obtained from a single patient will be assayed in the same batch to avoid inter-assay variability. The DLMO, a marker of circadian phase, is determined as the time point where melatonin production rises [57] using the hockey-stick method [58]. Patient-reported sleep data is used to calculate sleep midpoint. From sleep midpoint and DLMO, we can calculate the Phase Angle Difference (PAD) as a measure of entrainment [59]. The normal value of PAD is 6 h [56]. We expect patients to have a phase delayed DLMO relative to sleep midpoint with a PAD of approximately 5 h.

### Ranking of outcomes

The primary outcome is the change in interviewer rated levels of depression (blinded) measured by the Hamilton Depression Rating scale 17-item version. The secondary outcome is the change in self-assessed depression level based on the scores on the Major Depressive Inventory. The tertiary outcome is the change, in minutes, of the Phase Angle Difference (PAD) on the subset of patients assessed with Dim Light Melatonin Onset (DLMO).

Explorative outcomes are: (a) changes in quality of life based on changes on the WHO-5 wellbeing scale, (b) self-rated depression severity from the MDB system (c) self-rated sleep quality from the MDB system, (d) chronotype from the MEQ and, (e) sleep quality assessed by the Pittsburgh Sleep Quality Index, (f) Fitbit pulse and activity data.

#### Participants timeline

**Fig. 3:**
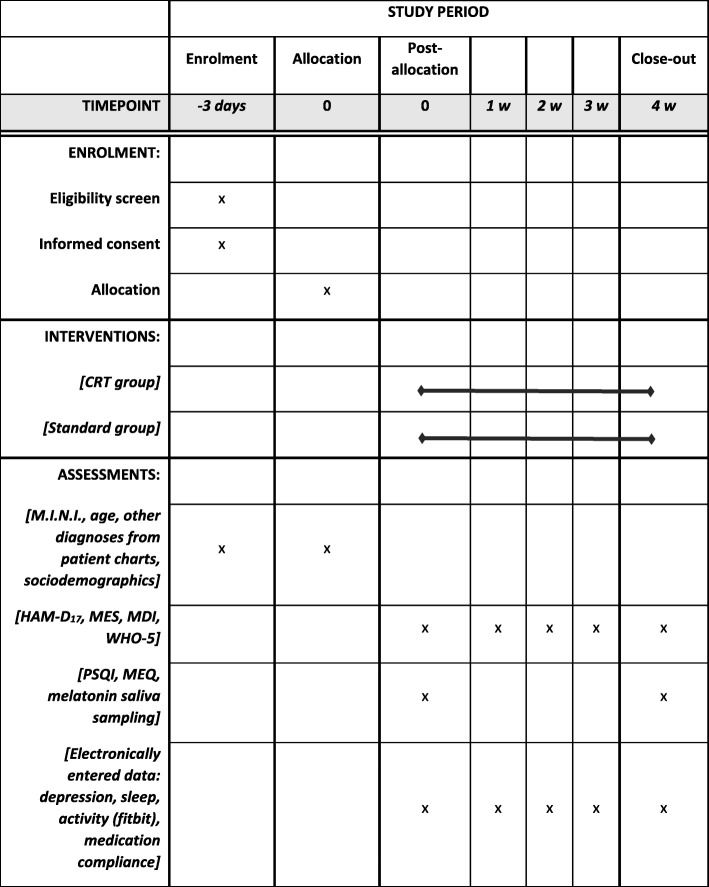
SPIRIT flow chart of study procedures

Participants are included while still on the inpatient ward or at latest 3 weeks after initiating treatment at the IOS and are followed for 4 weeks (see Spirit flow diagram in Fig. 3).

#### Sample size

Sample size calculations were done using the SAS 9.4 software.

The sample size for the primary outcome was calculated from an expected HAM-D_17_ mean score of 18 at baseline, a decrease to a score of 13 in the *Standard group* arm and to 10 in the *CRF group,* an expected pooled standard deviation of 6, an alpha level of 0.05 and a power of 0.80: a total of 128 participants is needed.

The sample size for the secondary outcome was calculated from an expected MDI mean score of 27 at baseline, a decrease to 22 in the *Standard group* and to 16 in the *CRF group*, with an expected pooled standard deviation of 12, an alpha level of 0.05 and a power of 0.80: a total of 128 participants will be needed.

The sample size for the tertiary outcome was calculated from an expected PAD of 5 h in the *CRT group* at baseline (a phase delay) normalizing to 6 h at endpoint, an unchanged PAD of 6 h in the *Standard group*, a PAD standard deviation of 80 min, an alpha level of 0.05 and power of 0.80: a total of 58 patients will be needed. We will collect saliva samples in 58 consecutive patients from participant randomization number 72.

#### Recruitment

When the IOS receive a referral, the attending physician will pass on information from the patient’s case file to the investigator, regarding age, dementia, psychotic episodes, and bipolar illness. If no exclusion criteria are present, investigators will contact the referring ward.

## Methods (assignment of interventions)

### Allocation

The randomization list was created from a computer randomization solution (http://www.randomization.com), by a person without any other connection to the trial. In all, 152 randomization numbers have been created allocating into either the *CRT group* or the *Standard group*, with no stratification factors. The randomization list is only accessible to the investigator responsible for randomization. The randomization sequence has been transferred to code-letters, stored in opaque envelopes in a locked cabinet. Block size is blinded for investigators. Primary investigator will enroll and assign participants to interventions. The patient will be allocated to the next study number and given an opaque allocation envelope containing information of trial group allocation: *Standard group* or *CRT group*.

### Blinding

The rater performing the Hamilton interview is blinded to treatment allocation and has no other association with the study. We do not consider it necessary in any circumstances to reveal a participant’s allocated intervention during the trial.

## Methods (data collection, management, and analysis)

### Data collection

All assessments are carried out by psychometrically trained investigators (ASA, KM). The Hamilton ratings are conducted by a certified Hamilton rater blinded for study group allocation (AP). The patients are instructed not to reveal group allocation to the rater. Trial duration is 4 weeks, including baseline assessment (pre-intervention) and final assessment (post-intervention). Patients not entering data into the MDB system for 2 days will be contacted by phone. Patients not showing up at endpoint visit will be contacted for a new appointment. Only patients with an endpoint Hamilton assessment are considered as completers.

### Data management

Data from the MDB application is exported from the website after each completed patient, as an excel file. All data files are stored on a secure logged server approved by the Danish Data Protection Agency. Paper and pencil records are stored in a locked room in a locked cabinet. All data will be checked for range and missing values.

### Statistical methods

Sociodemographics, scale scores, self-assessed outcomes, Fitbit data, and DLMO timepoints, will be described by summary and frequency statistics. Comparisons between groups will use parametric or non-parametric analyses according to normality distribution of data. Self-assessed daily data (depression, activity, and sleep) will be analyzed within a Mixed Model Repeated Measures analysis with a Random-effects Regression Model (RRM) [60]. Hamilton scale scores will be analyzed in a general linear model with baseline control. No interim analyses are planned.

## Methods (monitoring)

### Data monitoring and auditing

According to Danish law, only drug trials are required to comply with Good Clinical Practice guidelines. Data analyses will be supervised by a trained statistician. The project is subject to auditing from the regional ethical committee.

### Harm

We do not expect that the study will expose any participants to serious adverse advents or reactions as their usual clinical management is maintained. All adverse events will be recorded and rules for reporting of serious adverse events and serious adverse reactions will be adhered to. Any harm due to the study procedures is covered by the Danish Patient Compensation Association.

## Ethics and dissemination

### Research ethics approval

The study was approved by The Regional Committee on Health Research Ethics (H-15013943) and the Danish Data Protection Agency (2012–58-004/ RHP-20016-0016). All participants will provide a signed informed consent before enrollment into the study. Informed consent will be obtained by the primary investigator. The project will be stopped if participants develop serious side effects or if the MDB system becomes unavailable. Patients can leave the study at any time at their own discretion.

### Consent or assent

The staff at the inpatient ward, or at the IOS, will ask the referred patient whether to consent to an information meeting about the study. The patient will receive written and oral information regarding the trial from an investigator. The patient will be asked to decide on participation within 2 days. If the patient accepts, the informed consent will be signed, and the inclusion and baseline assessments will begin.

### Confidentiality

Patient confidentiality will be secured by anonymous participant identification on all pen-and-paper sheets and in electronic files. The participant identification list is stored on a secure, password protected server, only accessible to investigators. MDB data are stored on a secure logged server where all data is secured by a unique identifier with data entry logs and edit trail.

### Access to data

The final data set will be accessible to all persons in the project group. Data analyses will be supervised by a qualified statistician.

### Dissemination policy

Results will be communicated as papers in peer-reviewed international journals, as posters, and as oral presentations at international symposia. All data whether negative, positive or inconclusive will be reported in full. All members of the project group are co-authors with a pre-arranged order. No professional writers will be used. Depending on the journal of publication, part of the protocol, statistical code, and dataset will be publicly available.

## Discussion

In the review by Van’t Hof from 2009, several advantages and disadvantages for electronic self-help interventions [61] are listed. These include: saving therapist time, letting the patients work at their own pace in their own home, avoiding social stigma by attending psychiatric services, can function as a first step to enter treatment, securing the anonymity of the patient, monitoring patient progress more closely. A number of disadvantages are listed including: the inability of self-help programs to take care of comorbidity, the clinical information based on non-verbal and verbal clues are lost, systems do not allow a proper diagnostic interview making misclassification and wrong treatment a risk, lack of response to complications during treatment unless the system includes clinician feedback, and some patients might not be able to handle electronic systems.

Our own experience with these systems, when used as a tool in depressed patients in an inpatient ward, is quite similar [6]. Electronic self-monitoring can give flexibility for patients that can use the programs whenever it suits them, patients can use the electronic self-monitoring system to prepare for a consultation with a psychiatrist, and progress or worsening can be co-monitored by patient and clinician and adequate steps can be taken earlier. Disadvantages are lack of face-to-face interactions, the risk of missing important symptoms that are not monitored, nonverbal communication between patient and psychiatrist is missing, no assessment of psychomotor inhibition and mood reactivity and some patients find it difficult to handle the electronic systems which can increase feelings of worthlessness.

The design of this trial is not a standard RCT with placebo control, but rather usual care with two different add-on treatments: electronic monitoring in the *Standard group* and electronic monitoring and Circadian Reinforcement Therapy in the *CRT group*. Participants might gain from participating in both treatment groups from using the MDB system that enables them to gain insight into their symptoms making some sort of empowerment likely. This might limit the possibility to show any superiority of the *CRT* method in these patients with more severe depression, where improvement is very slow. The effect of the *CRT* method has not previously been tested, and we cannot be certain that the CRT intervention will aid patients in the transition phase from inpatient to outpatient status. We have concerns whether patients in this phase are too burdened by depression symptoms to be able to integrate new knowledge and habits into their daily lives. Regarding the electronic monitoring system, our experience is that any procedure in this phase needs to be very user-friendly and adaptable. We have designed the MDB system to comply with these conditions, but we cannot know to what extent these patients can manage to self-assess their state and look at the graphical interface to deduce patterns and make changes to their daily activities and sleep habits.It is a limitation of the trial that we only include patients having been admitted to an inpatient ward. The reduces generalizability, as we cannot know whether patients with less severe depression might react differently to the intervention. It is also a limitation that the trial only covers a period of 4 weeks. Finally, the technology has already advanced since the design of the MDB system and currently, the direction is towards app-based systems that can be used directly on Smartphones.

We hope that the *CRT* in combination with the MDB system will prove to be an efficacious new non-pharmacological treatment for patients with depression building on empowerment by giving patients new monitoring tools, physiological and psychological insights, the opportunity to be more involved in their own treatment, and more access to clinicians. If the *CRT* intervention proves successful it might be used for longer durations to give more a sustained impact.

## Abbreviations

CRT: Circadian Reinforcement Therapy
DLMO: Dim Light Melatonin Onset
DSM-IV: Diagnostic and Statistical Manuals of Mental Disorders
HAM-D_17_: Hamilton Depression Rating Scale 17 item version
ICD-10: International Classification of Diseases 10th revision
IOS: Intensive Outpatient Service
M.I.N.I.: Mini International Neuropsychiatric Interview
MDB: Monsenso Daybuilder System
MEQ: Morningness-Eveningness Questionaire
MES: Bech-Rafaelsen Melancholia Scale
PAD: Phase Angle Difference
PSQI: Pittsburgh Sleep Quality Index
RCT: Randomized controlled trial
SCN: Suprachiasmatic nuclei of the hypothalamus
